# FIB-milled plasmonic nanoapertures allow for long trapping times of individual proteins

**DOI:** 10.1016/j.isci.2021.103237

**Published:** 2021-10-08

**Authors:** Wayne Yang, Madeleine van Dijk, Christian Primavera, Cees Dekker

**Affiliations:** 1 Kavli Institute of Nanoscience, Delft University of Technology, Delft, The Netherlands

**Keywords:** Physical chemistry, Biophysical chemistry, Protein, Materials science

## Abstract

We have developed a fabrication methodology for label-free optical trapping of individual nanobeads and proteins in inverted-bowtie-shaped plasmonic gold nanopores. Arrays of these nanoapertures can be reliably produced using focused ion beam (FIB) milling with gap sizes of 10–20 nm, single-nanometer variation, and with a remarkable stability that allows for repeated use. We employ an optical readout where the presence of the protein entering the trap is marked by an increase in the transmission of light through the nanoaperture from the shift of the plasmonic resonance. In addition, the optical trapping force of the plasmonic nanopores allows 20-nm polystyrene beads and proteins, such as beta-amylase and Heat Shock Protein (HSP90), to be trapped for very long times (approximately minutes). On demand, we can release the trapped molecule for another protein to be interrogated. Our work opens up new routes to acquire information on the conformation and dynamics of individual proteins.

## Highlights

• We demonstrate fabrication of arrays of inverted-bowtie-shaped plasmonic gold nanopores
• Arrays (>64) of bowties with 10 to 20-nm size gap and single-nanometer variation can be produced
• We optically tweeze and detect single 20-nm polystyrene beads and individual proteins
• Our system allows for long observations (approximately minutes) of protein dynamics

## Graphical abstract

**Figure:**
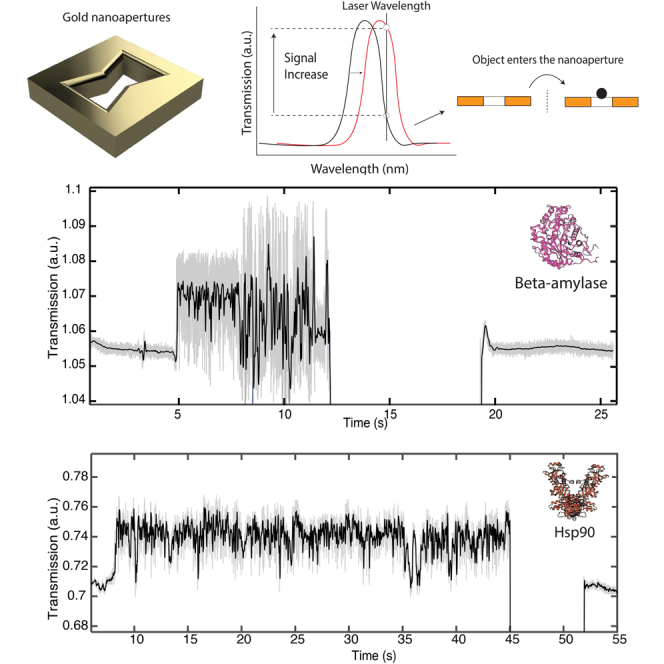

## Introduction

Proteins are responsible for virtually all cellular functions (Ha, 2014; Moerner, 2007). Just to name some examples that we will explore in this work, beta-amylase is a catalytic enzyme that allows cells to breakdown and digest glucose (Kaplan et al., 2006; Hopkins et al., 1948), whereas Heat Shock Protein is a chaperone protein that assists the folding of many other proteins into their proper functional shape (Whitley et al., 1999). Many proteins are molecular machines that can undergo various physical shape changes while performing their function. To understand the underlying mechanism of such conformational changes, it is necessary to resolve the internal dynamics at the single-protein level. Traditional techniques such as X-ray diffraction and cryo-EM resolve protein structure at near-atomic-level resolution but yield only static images, where dynamics has to be surmised (Cusack et al., 1998; Abola et al., 2000; Jonic and Vénien-Bryan, 2009; Fernandez-Leiro and Scheres, 2016; Cheng, 2018; Jackson, 2012). Other techniques such as observing fluorescence resonance energy transfer (FRET) require site-specific labeling of the proteins, which may interfere with their function (Ha et al., 2012; Joo et al., 2008). Indeed, there has been a strong drive in biophysics to search for new label-free single-molecule tools that are capable of sensing protein dynamics *in vitro* (Moerner, 2002).

An appealing idea is to trap a single protein, label-free and tether-free, in an aqueous buffer for an extended period of time for observation of its internal dynamics (Bustamante et al., 2020; KC Neuman, 2008; Maragò et al., 2013; Chen et al., 2012). One approach has been to use solid-state nanopores where a nanoaperture is etched through a SiN membrane (Yusko et al., 2011, 2017; Movileanu, 2009). By monitoring the ionic current through this nanoscale opening, one may attempt to obtain some information on the protein shape and conformation as the protein translocates through the nanopore (Willems et al., 2019; Japaridze et al., 2021). However, there are fundamental limitations in the sensing time as characteristically, the translocation time of the analyte is very fast (in the range of microseconds to milliseconds) (Plesa et al., 2013). Hence, such approaches are limited to capturing only snapshots of the protein shape. “Electrostatic fluidic traps” are an interesting alternate approach where an array of 200-nm-deep indentations is made onto a substrate such as SiO_2_ to form wells (Mojarad and Krishnan, 2012). The walls of the wells pick up an electrical charge in solution that repels biomolecules that are like-charge. This creates trapping regions in the wells for the biomolecules located in the center of the wells. This method has been shown to be able to distinguish differences in the distribution of electrical charges arising from different protein conformations (Ruggeri et al., 2017). However, such traps require fluorescence labeling of the protein, stochastic diffusion of the protein into the traps, and very low salt concentrations that are well below physiological concentrations. Newer techniques, such as the NEOtrap, which traps a protein using an induced electroosmotic current rather than an electrophoretic force, are able to very significantly increase the trapping times (Schmid and Dekker, 2021), but the use of an ionic sensing method prevents parallelization for which electrical isolation of each sensor is needed. Various groups have proposed other different techniques, most notably, the Anti-Brownian Electrokinetic Traps (ABEL) that tracks the molecule of interest by applying a real-time electrokinetic feedback to compensate for the drift from Brownian motion (Cohen and Moerner, 2006; Cohen and Moemer, 2005; Fields and Cohen, 2011). The analyte can be kept in the field of view and monitored for seconds. However, this technique still requires the molecule to be labeled with a fluorescent tag in order to be accurately tracked.

Plasmonic nanostructures have gained significant interest for biosensing (Mejía-Salazar and Oliveira, 2018). These are nanostructures that are typically patterned into a material (such as gold or silver) that supports plasmons, coherent electron oscillations that are driven by incident light (Maier, 2007). These structures have the ability to focus electromagnetic fields into nanoscale volumes below the diffraction limit. The optical resonance of these structures is extremely sensitive to the geometry of the structures and the local dielectric environment at subdiffraction volumes (<nm^3^). One class of these structures are plasmonic nanopores, nanoapertures that are through-holes that are milled through the freestanding metal layer that support plasmon excitations (Gordon et al., 2008; Garoli et al., 2019; Dahlin, 2015). These structures can exhibit an enhanced optical transmission (extraordinary transmission), which can be magnitudes higher than classically predicted. An analyte entering this volume will cause a shift in the optical resonance that can be probed with a high-speed photodetector (typically an avalanche photodiode, APD). By monitoring the transmitted light at a single wavelength, an induced shift in the optical resonance of the nanoaperture will lead to a measurable change in the transmission level recorded on the detector.

In addition, the tight concentration of the incident laser light by the nanoaperture, in particular across a gap within the nanoaperture, can produce strong optical gradient forces that can give rise to a tweezing force (Roxworthy et al., 2012; Gordon et al., 2008; Chen et al., 2012). These have been demonstrated for a variety of nanoapertures that were shown to tweeze small dielectric nanoparticles, i.e., beads and even single proteins. For example, Gordon et al. used nanoapertures that are supported on a glass substrate to detect and tweeze polystyrene (PS) beads and proteins (Kotnala and Gordon, 2014; Pang and Gordon, 2011). The majority of trapping times of these structures have so far been limited to a few seconds, before the proteins would spontaneously escape due to Brownian motion. If further developed to longer trapping times, this technique may potentially be used to detect changes in the shape of the proteins, as different conformations within the nanoaperture may show up as different levels in the optical transmission (Kotnala and Gordon, 2014; Zehtabi-Oskuie et al., 2013). Most developments have focused on these well-shaped traps with one opening closed off by the substrate. However, the development of a through-hole nanoaperture does allow for features such as electrophoretically defined directionality and ionic sensing to be integrated, allowing for full control over the analyte's motion instead of relying on diffusive processes to drive the molecule into the sensing region as well as having the benefit of two complementary sensing techniques, viz., optical and electrical (Verschueren et al., 2019a, 2019b; Shi et al., 2018). However, general progress on this tweezing and sensing technique has been hindered by challenges in the fabrication of plasmonics nanoapertures, as minute variations in the geometry of the fabricated structures give rise to large differences in the optical and nanotweezing properties. Fabrication of consistent plasmonics nanoapertures with reliable optical characteristics will open up avenues for high-throughput parallel optical sensing across the entire field of view on one device.

Here, we demonstrate a focused ion beam (FIB) method to reliably fabricate an array of bowties on a single membrane with outstanding properties for single-molecule trapping. The nanoapertures were defined in an inverted bowtie shape and milled into a layer of Au. We characterized the geometry by the gap size and the length of the bowtie. The variation in the two critical dimensions of the produced geometries within a produced array were found be very small, i.e., less than 2 nm. Consistent with the shape homogeneity of the plasmonic structures, all nanoapertures in the array displayed a very similar optical response in terms of their optical transmission. The traps were used to demonstrate first data of nanotweezing of two types of proteins (beta-amylase, HSP90), which sometimes could be stably trapped for very long times (>3 min). Proteins could be released on demand, and the structures were robust enough to be used across multiple experimental runs. Taken together, these structures show potential for probing single-molecule protein dynamics.

## Results

### Fabrication and transmission electron microscopy characterization of inverted bowtie structures

We fabricate our plasmonic structures using direct FIB milling in a gold layer. Most fabrication methods for the production of nanoapertures are based on e-beam lithography and etching. By contrast, FIB milling uses a highly energetic beam of ions (typically Ne or Ga) to knock out atoms directly from the substrate (Giannuzzi and Stevie, 2004). The lack of a masking step that is required in standard e-beam lithography techniques eliminates variables such as the resolution of the resist mask, development times, etching times, and residual polymer remnants, which can drastically vary and thus influence the final shape of the produced structures (Henzie et al., 2009). FIB milling simplifies the process and reduces the parameters to two variables, the spot size and dwell time of the ion beam used.

**Figure 1:**
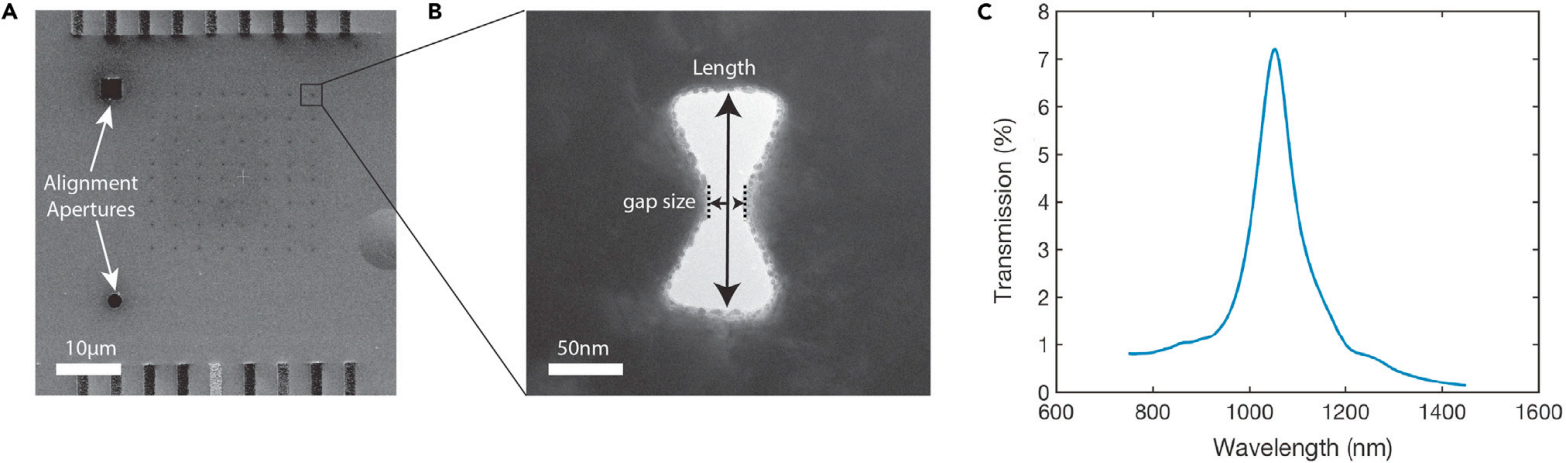
Array of inverted bowtie nanoantennas (A) SEM image of an array of inverted bowtie antennas. The apertures were drilled into a 100-nm-thick gold film on a freestanding SiN membrane by local milling with a Focused Ion Beam (FIB). Two alignment apertures were milled next to the array. (B) TEM image of FIB-milled nanoaperture. The length and gap size are indicated. The fabricated structures were very consistent across the array with an average length and gap size of 160 ± 2 and 23 ± 2 nm, respectively. (C) Finite-difference time-domain (FDTD) calculations for the transmission of bowtie as a function of wavelength, showing a peak at 1,055 nm, which is just below the laser wavelength of 1,064 nm.

Plasmonic structures were fabricated in a gold layer that was deposited onto a freestanding SiN membrane. A thin adhesion layer of 2–3 nm of Ti was deposited onto the SiN, before 100 nm of Au was deposited, which provided the starting material for the plasmonics aperture to be milled in. We chose to fabricate inverted bowtie-shaped structures where the electromagnetic field will be concentrated at the edges near the center of the bowtie. Such an inverted bowtie shape also provides good thermal properties as heat from the incident laser is drained to the surrounding gold, which acts as a heat sink. The structures were drilled with a Ga ion beam (30 KeV) by intersecting two overlapping inverted triangles (Chen et al., 2016). The dose and amount of overlap were optimized to produce the desired result in the gap size. The geometry and final gap size were found to be strongly dependent on the spot size of the ion beam aperture used. Figure 1 shows an example of a SEM image of an array, as well as a transmission electron microscopy (TEM) image of a fabricated bowtie. Here, we fabricated 8 × 8 inverted bowties with the same parameters (Figure 1A). The bowties were mutually spaced 2.5 μm apart in order to ensure that only one bowtie was illuminated within the laser spot (∼1 μm) during the optical measurements. In addition, we milled two larger apertures for the alignment and calibration of the laser.

The dimensions of the fabricated structures were characterized under TEM (Figure 1B). For this array, the size of the gap was found to be 23 ± 2 nm (mean ± SD), whereas the length of the inverted structure was 160 ± 2 nm (mean ± SD) (N = 12). The bowties measured were taken along the diagonal of the array (from top left to bottom right) with four more bowties taken along the opposite diagonal. Across fabrication runs, the measured SD did not differ and ranged from 1 to 2 nm. At the edges of the structures, we observed some grains from the deposited gold after milling, which appeared as jagged edges, but optical characterization showed that these had only a minor effect on the plasmonic properties.

### Optical characterization shows that the plasmonic nanoapertures are highly uniform

Finite-difference time-domain (FDTD) simulations were performed to calculate the transmission of the nanoaperture as a function of incident wavelength between 750 and 1,450 nm. Using the shape as determined from TEM images, we obtained a peak in the transmission, see Figure 1C. The results confirm that the structures are expected to have a maximum transmission peak in the near infrared region at ∼1,055 nm, i.e., just below the wavelength of the laser (1,064 nm) (Sullivan, 2013).

**Figure 2:**
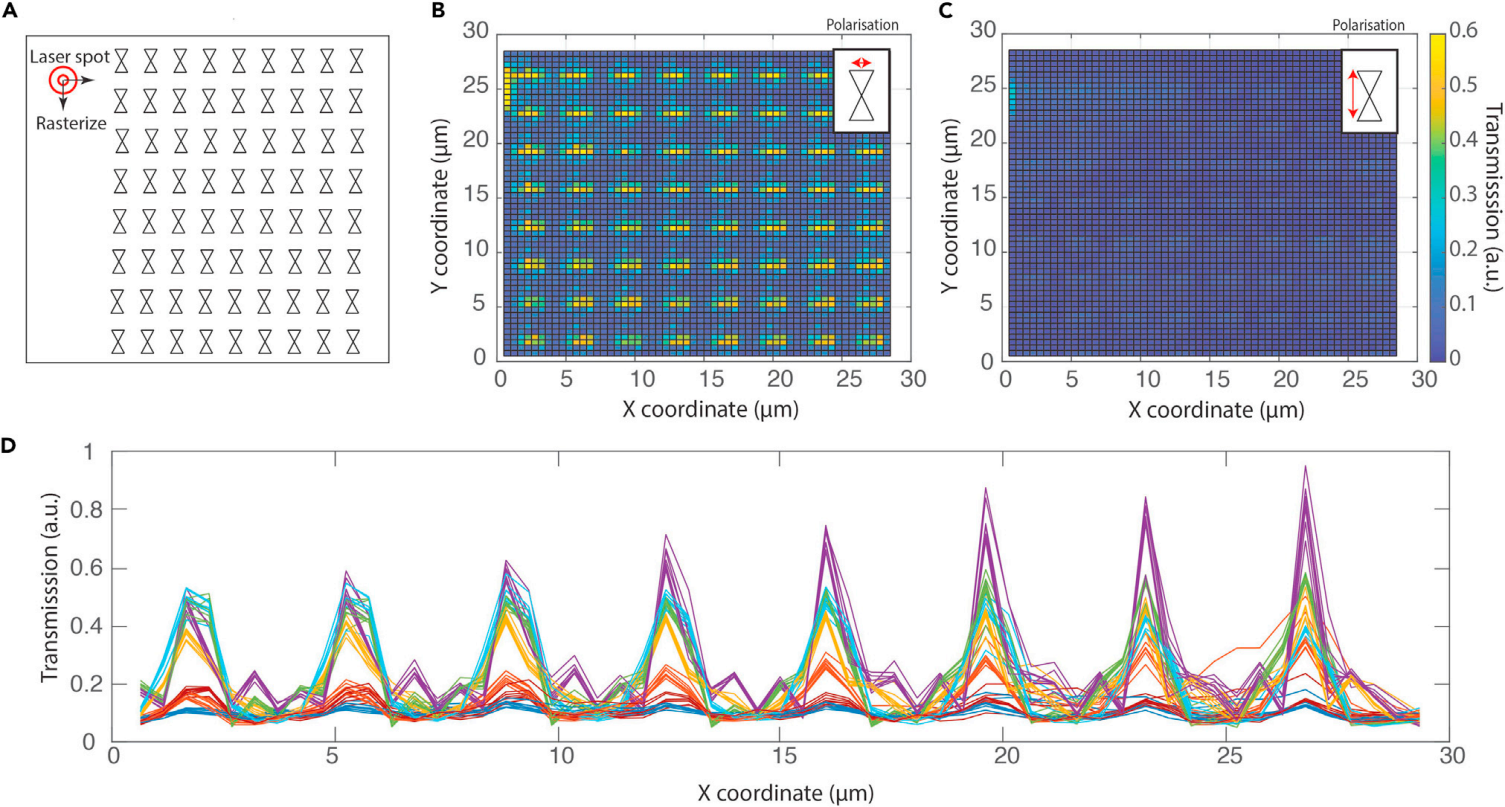
Characterization of optical transmission of inverted bowtie nanoantennas (A) Schematic of an array of inverted bowties. A laser can be raster scanned across the array in steps of 500 nm (i.e., with five steps to pass the mutual distance between bowties of 2.5 μm), and the transmission of each bowtie antenna is measured. Data were recorded for two orthogonal polarizations. (B) Heatmap of the optical transmission when the laser polarization was in the longitudinal polarization (across the gap). The measured transmission increased greatly when the laser was located at an aperture. For an example of how the transmission is affected by a larger gap size, see SI. (C) Same heatmap of the optical transmission when the polarization was orthogonal (across the length of the bowtie). For this polarization, the optical transmission remained low. (D) Line profiles of the measured optical transmission across rows of the array. Each row of the measured optical transmission in B was converted into a line profile and plotted in (D) The transmission is seen to increase to a high value (0.5–0.8) each time that the laser was positioned near a bowtie position. In the areas in between the bowties and in rows where the laser did not cross a bowtie, the optical transmission remained low (<∼0.2). Toward the bowties on the right side of the membrane, there was a small increase in the measured transmission because the large freestanding SiN membrane was not mounted entirely flat, which led to small changes in the alignment with the detector.

For measurement of the optical transmission of the bowtie apertures in the arrays, the sample was mounted in the laser setup (1064 nm, 20 mW) and an APD was used to collect the transmitted light. The laser was raster scanned across the array and the transmitted light was recorded for each bowtie and plotted in a 2D heat map (Figure 2A). We acquired such a 2D map for two different polarizations (transverse, along the length of the bowtie; and longitudinal, across the gap of the bowtie). Figures 2B and 2C show the 2D maps in the two different polarizations. For the longitudinal polarization, the transmission value increased drastically when the laser co-aligned with the nanoapertures. In areas between bowties, however, only a negligible amount of light was collected demonstrating that the thickness of the gold (100 nm) acted as a sufficient stop to the incident light. When the polarization was rotated by 90°, the transmission value dropped to the baseline, i.e., no extraordinary transmission of light occurred, as expected. We compared the optical transmission properties of the bowties by plotting each row of the 2D map as a line profile and overlaying them in Figure 2D. As seen from this overlay, the bowties yielded very similar optical transmission intensities with maximum values that were very similar for different bowties.

**Figure 3:**
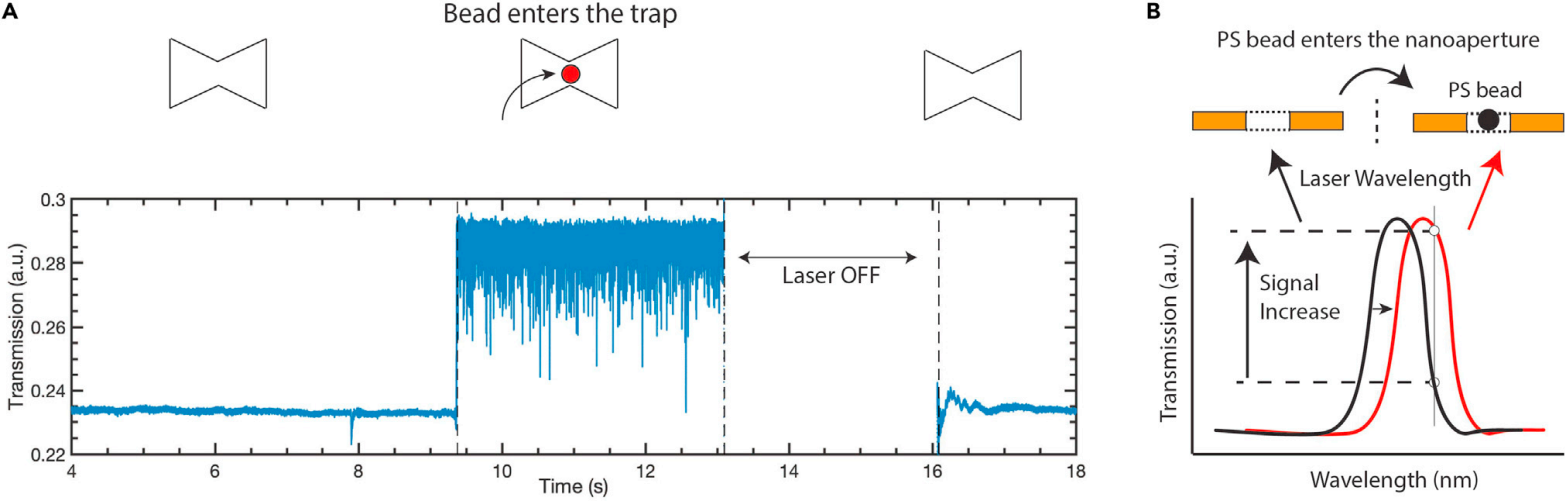
Optical trapping of 20-nm polystyrene beads (A) Transmission level signal versus time. The signal abruptly increased indicating that a single PS entered the trap. When the laser was temporary shut off and switched back on, the transmission level returned to the previous baseline indicating that the object had been released. (B) Schematic representation of the redshifting of the nanoaperture in the presence of a bead. The peak of the resonance redshifted toward the wavelength of the laser, which led to an increase in transmission of the nanoaperture.

To demonstrate the optical-trapping abilities of these plasmonic structures, we first performed control experiments with 20-nm PS beads (N = 46). In a liquid cell, we added PS beads (0.05% w/v) to the gold side of the sample. The laser was kept focused on a single inverted bowtie and the transmission level was recorded. After waiting for some time, usually a few tens of seconds, a sudden increase of the optical transmission was recorded for the bowtie. Figure 3A shows an example. Although the transmission level was initially stable, it suddenly increased at some point. This increased transmission was recorded for a few seconds, after which the laser was shut off. When, 3 s later, the bowtie was probed again, the transmission level had returned to the original level. No such traces were ever seen for buffers without PS beads. We interpret the increase in the signal as the optical trapping of a PS bead into the bowtie, which subsequently escaped the trap by diffusion when the laser was shut off. Upon entry of the PS bead into the trap, the optical resonance of the nanoaperture red-shifted toward the 1,064 nm wavelength of the laser, leading to an increase in the transmission level of the nanoaperture, as illustrated in Figure 3B.

### Trapping of single proteins

**Figure 4:**
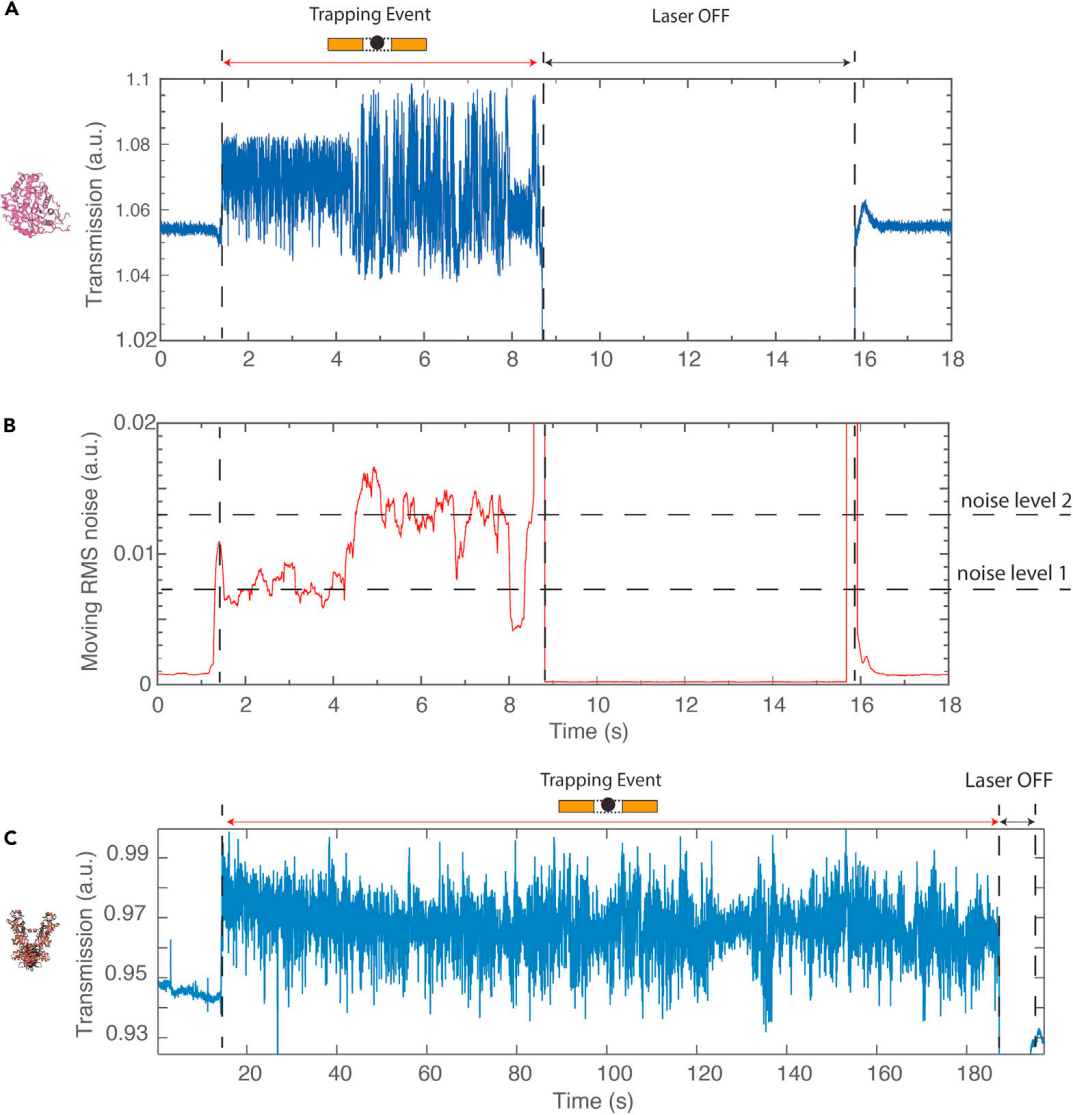
Optical trapping of single proteins (A) Transmission signal over time of a trapped beta-amylase protein. Similar to the PS bead in Figure 3, an increase in transmission was observed when the protein entered the trap. The transmission level returned to the baseline when the laser was turned back on after ∼5 s (B) Root Mean Square (RMS) noise of the trapping signal over time. We observe three levels of fluctuations: (1) low fluctuations for the empty trap, (2) an increased level of fluctuations upon trapping (noise level 1), and (3) an even higher level of fluctuations appearing at t = 4 s (noise level 2). The level decreases back to level ii at t = 8 s. There is a short increase back to noise level 2, just before the laser was turned off. (C) Example of a long trapping trace of HSP90. We successfully tweezed the protein for 3 min. After that, the laser was switched off for 10 s and turned back on. The baseline then returned to a low transmission level, indicating that the protein had been released.

Next, we explored the application of our plasmonic nanoapertures for the trapping and studying of individual proteins. We started with beta-amylase, a globular tetrameric protein that consists of four monomer subunits with a combined molecular weight of 223.5 kDa that is commonly used as a molecular standard that is stable up to 60° at pH 6–8 (Cheong et al., 1995). We introduced beta-amylase in our traps (0.1% w/v) and recorded trapping events (N = 112). To enhance the trapping rate, a −250 mV bias voltage was applied to drive the protein into the trap. Figure 4A shows a typical example of a trapping event. Similar to the experiment for the PS bead, the transmission through the nanoaperture initially remained at the baseline value but suddenly increased. We interpret this signal as an indication of a single protein getting trapped into a nanoaperture. Control experiments without protein in solution did not yield such trapping events. Furthermore, nanoapertures that had gap sizes too small to fit the protein (i.e., <5 nm gap) did not yield stable trapping signals.

During the trapping of a single beta-amylase protein, the transmission signal did not merely exhibit a change in intensity but also showed distinctly different fluctuation amplitudes (N = 112), which we characterized by the Root Mean Square (RMS) noise. A moving standard deviation was calculated and plotted in Figure 4B. The empty trap showed low fluctuations (an RMS of ∼0.001 a.u.). Upon trapping, the level of fluctuations increased drastically to an RMS of 0.007 a.u. An even higher level of fluctuations appeared at t = 4 s where the RMS increased to a value of 0.013 a.u. At t = 8 s, the level decreases back to the first level, just before the laser was turned off, suggesting two states that are reversible, and also showing that the data are not consistent with protein denaturation in the trap. To check that these observations and the trapping signal did not simply indicate unspecific sticking of the protein to the trap, we shut off the laser to show that we can release the trapped molecule and return the trap to its initial empty state. Of 112 trappings, only 1 such event did not respond in this way to the interruption of the laser. For the data recorded at an applied voltage of 250 mV (N = 46), we sorted the percentage of time that the beta-amylase spent in various states, based on the RMS noise levels, i.e., at noise level 1, noise level 2, and others. Overall beta-amylase spent 53% of the time at noise level 1 and 30% of the time at noise level 2. The remaining duration (17%) was spent at an RMS noise level in between the two identified levels and could not be attributed unambiguously.

These different levels were not observed in any of the trapping trace obtained from trapping 20-nm PS bead and are unique to beta-amylase, suggesting that they represent different internal molecular states of the protein. One may speculate that this is due to fluctuations with the long C-terminal loop (from residue 445 to 493 of the peptide chain) found in each subunit of the tetramer that is proposed to interact with the secondary structure (Cheong et al., 1995). Partial unfolding due to heating is less likely since beta-amylase was found to be resistant to degradation and keeping its functionality up to 65° (Mensah et al., 2016). What is clear from the traces that we observed is that the states appear to be reversible with the protein trapping signal transiting between the two levels during the course of a trapping event.

Next, we studied HSP90 proteins (N = 12). HSP90 is a dimeric protein complex (total mass 162.8 kDa) that belongs the family of chaperone proteins that are important for proper folding and maintenance of other proteins within the cell (Jackson, 2012; Schmid et al., 2016, Schmid et al., 2021). They have been reported to undergo conformational changes between an open and a closed state that involve a 5- to 8-nm change in the length span of the protein. FRET studies, which require site-specific labeling of the protein, indicate that these transitions occur on the 0.1- to 0.5-Hz timescales (Hessling et al., 2009; Mickler et al., 2009; Richter and Buchner 2006).

Upon addition of 50 nM of HSP90 proteins to the system, we observed the same type of single-protein trapping events as for beta-amylase, with a sudden increase in the transmission signal, indicating that a protein was being tweezed; see Figure 4C for an example. The data indicate that very long trapping times could be realized, as can be seen in the example event, which shows a 3-min trapping. In practice, the trapping events were terminated by shutting down the laser to ensure that the proteins were not simply sticking to the trap.

**Figure 5:**
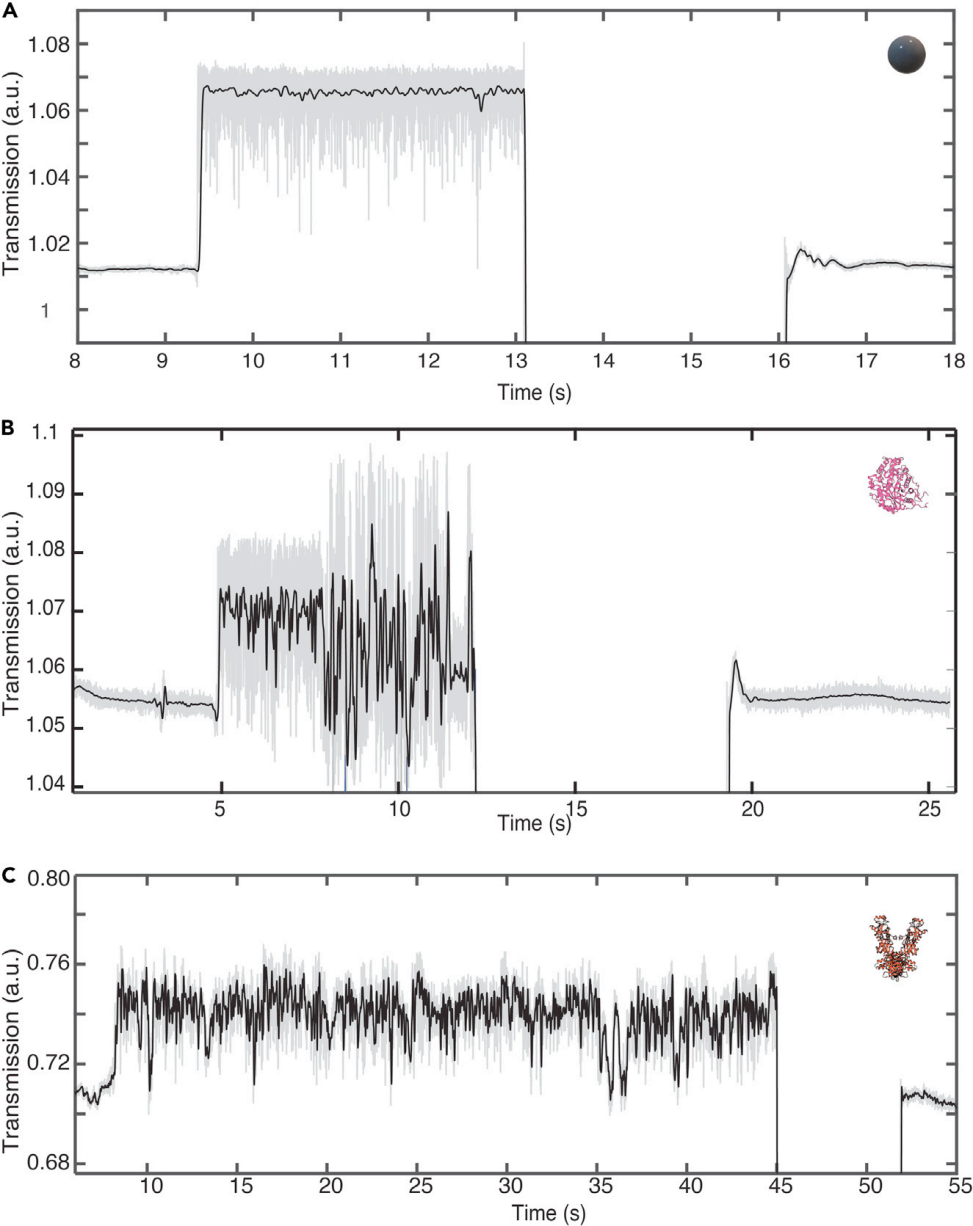
Effect of low-pass filtering on data traces Unfiltered traces are plotted in grayscale in the background, and 10-Hz low-pass-filtered traces are plotted in black (A) Data for a PS bead. Fluctuations in the transmitted signal for the PS bead reduced to a single homogeneous level when the signal was low-pass filtered. (B) Data for beta-amylase. (C) Data for HSP90. The low-frequency modulations are still present in the trapping signal for the protein traces unlike the PS bead trace, indicating significant low-frequency fluctuations.

The trapped proteins showed pronounced low-frequency fluctuations. To visualize this, we applied a low-pass filter of 10 Hz to the traces; see Figure 5. In the case of the PS beads, most of the fluctuations were filtered out, indicating that the major fluctuations involved higher characteristic frequencies. For the protein traces, however, substantial low-frequency fluctuations prevailed in the signals, most remarkably in the Hsp90 trapping trace. At this stage, it is not fully resolved what underlies these fluctuations. Intermittent sticking interactions with the surface is unlikely given that most of the proteins diffuse quickly out of the trap at the moment that the laser is shut off. Rotational diffusion of the proteins is another hypothesis but is expected at much higher frequency; e.g., Houghtaling et al. reported that the estimated rate for a 150-kDa protein is ∼10^6^ rad^2^ s^−1^ (Houghtaling et al., 2019). In our system we expect similar timescales for rotational diffusion of the protein and hence rule it out given our 10-Hz low-pass filter. Slow internal conformational changes are an attractive candidate for causing the low-frequency noise as the sub-hertz timescale matches the reported range for protein conformational changes (Schmid and Dekker, 2021), but further research is needed to rigorously establish this.

Interesting, we found that the nanoaperture devices were very robust in these protein trapping experiments. We successfully reused the same devices in multiple experiments. Between each experimental run, the sample was rinsed in H_2_O and ethanol, followed by an oxygen plasma (2 min, 50 mW) to remove organic contamination. In general, the same antenna could be used multiple times for observation times of hours. TEM inspection before and after a series of three experiments on the same bowtie yielded no observable changes to the nanoapertures such as due to protein residues.

### Conclusions and outlook

In conclusion, we demonstrate a direct FIB milling method to fabricate nanoapertures for optical sensing and tweezing. Our optical nanoaperture devices with a through-hole allow for integration with electrical readout methods and for the analyte to be driven electrophoretically in a user-defined direction instead of relying on diffusion. We explored the application of our plasmonic nanoapertures for the study of single proteins, where a variety of single proteins can be monitored for long observation times in a label-free manner. We were able to tweeze and trap proteins for extended periods of time (minutes) paving the way to study slow or infrequent conformational changes within a single protein. The proteins showed distinct low-frequency fluctuations in their transmission level, possibly associated with protein conformational changes.

Looking ahead, monitoring the kinetics of single proteins will open up new exciting avenues using plasmonics nanotweezers for the study of protein dynamics. More experiments are needed to tease out if it is possible to sense protein conformation and shape using label-free resonance shift sensing. A possible extension is to use the plasmonics nanoapertures to tweeze the protein in addition to using FRET labels to study protein conformation dynamics (Kajihara et al., 2006). Although this is an inviting perspective for single traps, parallelization may face technical challenges, such as sufficiently strong illumination by the laser across the entire sensor array, which may lead to laser-induced heating that causes protein denaturation (Lee, 1991).

Although these obstacles challenging, none of them are, however, insurmountable. All in all, we believe that plasmonics nanoapertures hold great promise for label-free interrogation of proteins on the single-molecule level for extended times.

### Limitations of the study

Specific instrumentation is needed as the fabrication protocol requires a Focused Ion Beam and fabricated traps were inspected by Transmission Electron Microscopy to ensure that they were still viable after every experiment. Further work needs to be done to deconvolute the various contributions to the dynamic signal (that may be due to e.g. protein conformations, rotational diffusion, and sticking). The orientation of the trapped proteins within the nanoaperture is not well known. Caution is called for in generalizing observations of proteins in *in vitro* data to *in vivo* applicability in cells.

## STAR★Methods

### Key resources table

**Table:** 

REAGENT or RESOURCE	SOURCE	IDENTIFIER
**Biological samples**		

β-Amylase from sweet potato	Sigma Aldrich	9000-91-3
Hsp90	Hugel ‘s lab	2CG9

**Software and algorithms**		

Matlab	Version 2019b	www.mathworks.com
Transalyzer	Calin Plesa	https://github.com/voyn/transalyzer
Nanobuilder	ThermoFisher	https://www.thermofisher.com/order/catalog/product/NANOBUILDER#/NANOBUILDER

**Other**		

Focused Ion Beam	FEI	Helios G4 CX

### Resource availability

#### Lead contact

Further information and requests for resources and reagents should be directed to and will be fulfilled by the Lead Contact, Cees Dekker (c.dekker@tudelft.nl).

#### Materials availability

This study did not generate new unique reagents.

#### Data and code availability

Any additional information required to reanalyze the data reported in this paper is available from the lead contact upon request.

### Method details

#### Fabrication of nanoapertures

The nanoapertures were fabricated in gold films on 20nm free-standing SiN membranes that were fabricated according to the protocol listed by Janssen et al. 1–3 nm Ti was sputtered on the substrate as an adhesion layer, after which 100nm of Au was deposited using an electron-beam evaporator (Temescal). The nanoapertures were then milled with a Ga beam in a FEI Helios G4 CX (30KeV, 2pA beam).

#### Experimental setup

The sample was first rinsed in ethanol and H_2_O and cleaned in an O_2_ plasma at 50W for 1 minute. The sample is mounted in a custom-made PEEK flowcell that allows for the laser to optically excite the nanoapertures and for the transmission light to be collected. The flow cell was either filled with H_2_O or with 1x phosphate buffered saline (PBS). There was no noticeable change in the optical transmission of the nanoapertures between the two different buffers as they have very similar refractive indexes. A 1064nm laser was focused onto the nanoapertures (M9-A64-0200 laser diode, Thorlabs) with a 60× objective. The laser light was collected using a 10×0.3NA objective (Nikon) and projected onto an Avalanche Photo Diode (APD410C/M Thorlabs). A 2D map was taken by raster scanning across the array of nanoapertures using a piezoelectric stage (MadCity Labs Inc). The transmission was acquired at a sampling rate of 200 kHz and low-pass filtered down to the indicated values.

#### Analytes

We studied the following analytes for the experiments: 20nm polystyrene beads (Thermo-Fisher)- 0.05% w/v; beta-amylase (Sigma) −0.05% w/v; Heat Shock Protein 90 (HSP90) (Obtained from the Hugel Lab)- 50 nMol. All analytes were diluted in 1X PBS that was additionally buffered with 5nM of MgCL in the case of the HSP90.

### Quantification and statistical analysis

#### Analysis of events

Event detection and analysis was performed using Tranzalyser, a custom-made MATLAB-based software package (Plesa and Dekker, 2015). All traces shown in the plot were low-pass filtered at 10 kHz unless indicated otherwise.
