# Supplementary material for: FIB-milled plasmonic nanoapertures allow for long trapping times of individual proteins
Source: iScience. 2021 Oct 8;24(11):103237. doi: 10.1016/j.isci.2021.103237 (PMC8551080; doi:10.1016/j.isci.2021.103237)
Supplement: Document S1. Figures S1–S3 [file mmc1.pdf]

iScience, Volume *24*

Supplemental information

FIB-milled plasmonic nanoapertures allow for long trapping times of individual proteins

Wayne Yang, Madeleine van Dijk, Christian Primavera, and Cees Dekker

Supplementary Information - FIB-milled plasmonic nanoapertures allow for long trapping times of individual proteins

Wayne Yang, Madeleine van Dijk, Christian Primavera, Cees Dekker
Kavli Institute of Nanoscience, Delft University of Technology, The Netherlands

September 29, 2021

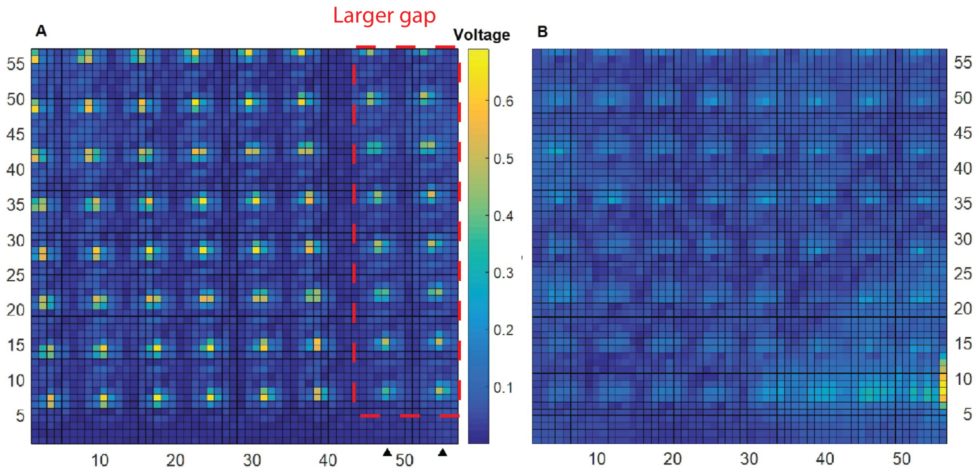

Figure S 1: **2D heat map of optical transmission of bowtie array in 2 orthogonal polarizations. Related to Figure 1 in main text.** The optical transmission of the array rose to the same optical transmission value, except for the last 2 columns that were slightly dimmer (red box). Notably the bowties in the last 2 columns were milled to have smaller gap sizes (2nm smaller).

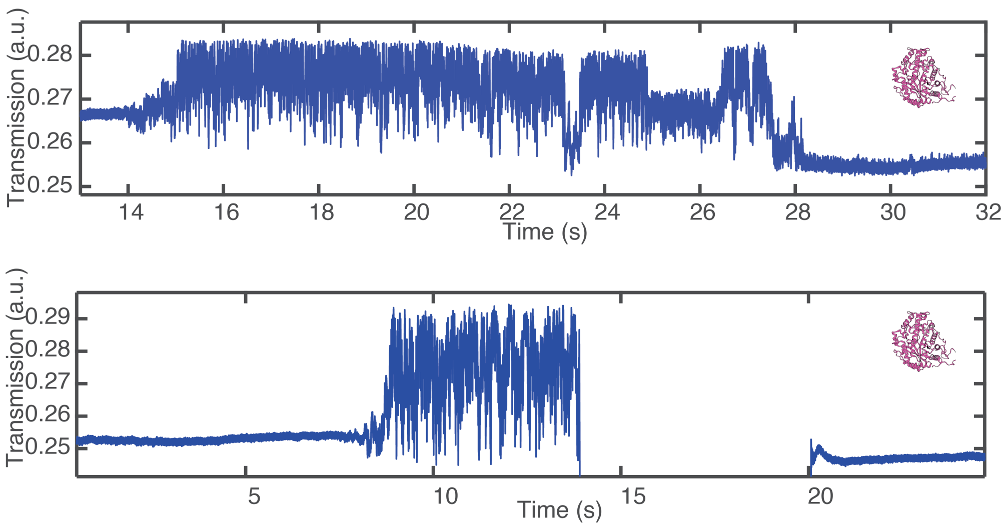

Figure S 2: **Additional beta-amylase trapping traces. Related to Figure 4 in main text.**

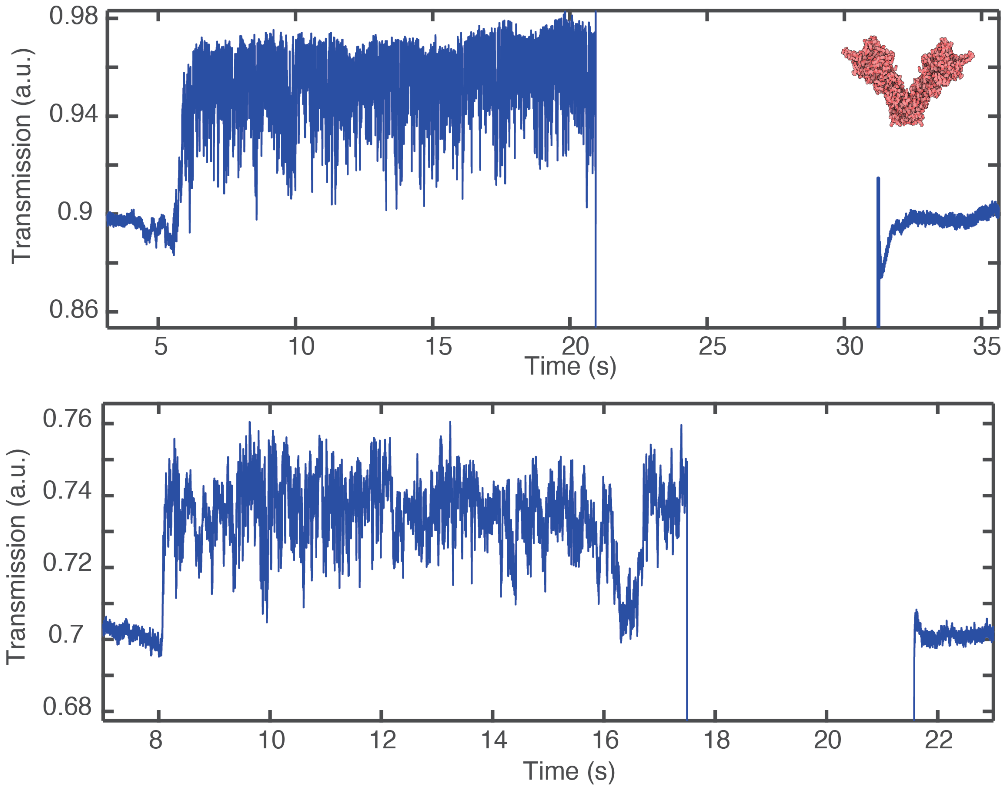

Figure S 3: **Additional HSP90 trapping traces. Related to Figure 4 in main text.**
